# Transcriptome Profile Reveals that Pu-Erh Tea Represses the Expression of Vitellogenin Family to Reduce Fat Accumulation in *Caenorhabditis elegans*

**DOI:** 10.3390/molecules21101379

**Published:** 2016-10-17

**Authors:** Ru-Yue Xiao, Junjun Hao, Yi-Hong Ding, Yan-Yun Che, Xiao-Ju Zou, Bin Liang

**Affiliations:** 1 Pharmaceutical College, Heilongjiang University of Chinese Medicine, #24Heping Road, Harbin 150040, China; xryvdld@163.com; 2 State Key Laboratory of Genetic Resources and Evolutionary & Functional Genomics, Kunming Institute of Zoology, Chinese Academy of Sciences, Kunming 650223, China; haojunjun@mail.kiz.ac.cn; 3 Key Laboratory of Animal Models and Human Disease Mechanisms of the Chinese Academy of Sciences & Yunnan province, Kunming Institute of Zoology, Chinese Academy of Sciences, Kunming 650223, China; dingyndl@163.com; 4 Pharmaceutical College, Yunnan University of Traditional Chinese Medicine, Kunming 650500, China; checpu@163.com; 5 Department of Life Science and Biotechnology, Key Laboratory of Special Biological Resource Development and Utilization of University in Yunnan Province, Kunming University, Kunming 650214, China

**Keywords:** Pu-erh tea water extract (PTE), fat accumulation, RNA-Seq, vitellogenin (*vit*) family

## Abstract

Due to misbalanced energy surplus and expenditure, obesity has become a common chronic disorder that is highly associated with many metabolic diseases. Pu-erh tea, a traditional Chinese beverage, has been believed to have numerous health benefits, such as anti-obesity. However, the underlying mechanisms of its anti-obesity effect are yet to be understood. Here, we take the advantages of transcriptional profile by RNA sequencing (RNA-Seq) to view the global gene expression of Pu-erh tea. The model organism *Caenorhabditis elegans* was treated with different concentrations of Pu-erh tea water extract (PTE, 0 g/mL, 0.025 g/mL, and 0.05 g/mL). Compared with the control, PTE indeed decreases lipid droplets size and fat accumulation. The high-throughput RNA-Sequence technique detected 18073 and 18105 genes expressed in 0.025 g/mL and 0.05 g/mL PTE treated groups, respectively. Interestingly, the expression of the vitellogenin family (*vit-1*, *vit-2*, *vit-3, vit-4* and *vit-5*) was significantly decreased by PTE, which was validated by qPCR analysis. Furthermore, *vit-1(ok2616)*, *vit-3(ok2348)* and *vit-5(ok3239)* mutants are insensitive to PTE triggered fat reduction. In conclusion, our transcriptional profile by RNA-Sequence suggests that Pu-erh tea lowers the fat accumulation primarily through repression of the expression of *vit*(vitellogenin) family, in addition to our previously reported (sterol regulatory element binding protein) SREBP-SCD (stearoyl-CoA desaturase) axis.

## 1. Introduction

When energy intake exceeds energy expenditure, the excessive energy is generally converted to fat accumulating in adipose tissue or non-adipose tissue, leading to obesity and obesity related diseases, such as type 2 diabetes (T2D), hyperlipidemia, fatty liver, hypertension, and insulin resistance [1,2,3]. Currently, obesity is one of the most common health concerns around the world, and its prevalence is increasing rapidly [4,5,6,7]. A recent analysis of trends in body mass index (BMI) has shown that the number of obese people has been growing amazingly in the past 40 years, and the number has been rising from 105 million in 1975 to 641 million in 2014 [8].

In order to fight against obesity, people have taken several approaches, such as a balanced diet, physical exercise, a healthy lifestyle, intake of functional foods, or pharmacological therapies [9]. Because medicines are often somewhat accompanied with side effects [10], natural products have been considered as safe therapies to lose weight, in recent years [11,12,13]. Tea is a popular beverage globally, and has been used as a medicine for more than 4000 years. Among a variety of teas, the fermented Pu-erh tea has been considered to have numerous health benefits, such as suppressing hyperlipidemia and hyperglycemia [14,15], lowering the atherogenic index [16], as well as anti-oxidation [17], anti-cancer [18], and anti-obesity in particular [19,20,21]. Pu-erh tea is produced from the leaves of tea trees (*Camellia sinensis* (L.) *var. assamica (Masters) Kitamura*) growing in Yunnan province, and fermented by microorganisms.

Although several studies, including our previous report, have shown that Pu-erh tea may target a few genes or signal pathways to reduce fat accumulation [15,18,22,23,24], the underlying mechanisms of its anti-obesity effect are still poorly understood, especially with a lack of a global view of genes expression at the genome level so far. RNA Sequencing (RNA-Seq) is a recently developed technique that uses massively parallel sequencing to analyze a comprehensive transcriptome of genomes [25]. With a high-throughput manner and a broad dynamic range [26,27], RNA-Seq has the capacity to measure individual gene expression accurately in order to identify novel regions of transcription [25], posttranscriptional modifications [28], and noncoding RNAs [29]. Therefore, we took the advantages of RNA-Seq to determine the gene expression profiling and transcriptional characters of the Pu-erh tea treated *C. elegans* and found that the *vit* family is responsive for Pu-erh tea’s function of reducing fat accumulation.

## 2. Results and Discussion

### 2.1. PTE Reduces Fat Accumulation and Modifies Fatty Acid Compositions

**Figure 1:**
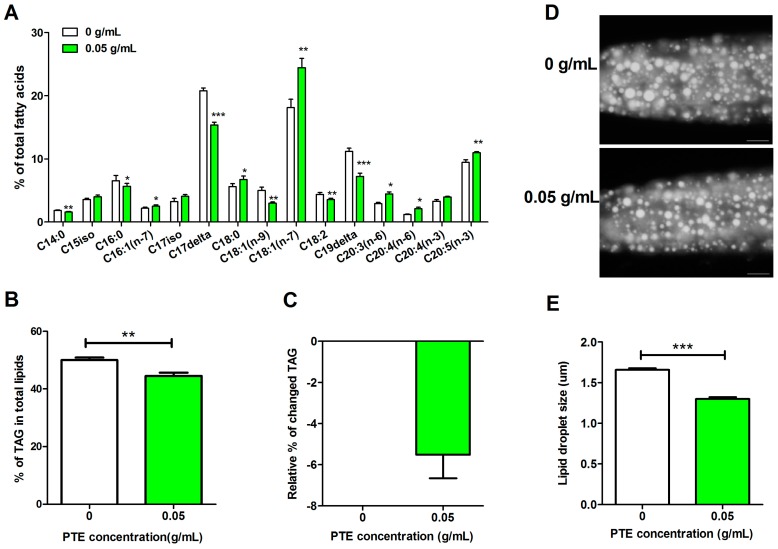
PTE reduced fat storage and modified the level of fatty acids compositions. (**A**) the level of fatty acids determined by thin-layer chromatography and gas chromatography (TLC/GC); (**B**) percentage of triacylglycerols (TAG) in total lipids (TAG + PL, phospholipids); (**C**) relative percentage of changed TAG; (**D**) Nile Red staining of fixed late L4 worms. Exposure time: 25 ms. Scale bar represents 20 μm. Data are presented as mean ± SEM of four biological independent repeats; and (**E**) quantification of lipid droplet size. Data are presented as ± SEM of 10 worms. Significant differences between PTE treated group (0.05 g/mL) and the control group (0 g/mL), *** *p* < 0.001, ** *p* < 0.01, * *p* < 0.05.

Consistent with our previous study [22], PTE indeed decreased fat accumulation and modified the fatty acid compositions in *C. elegans*. The levels of unsaturated fatty acids, including oleic acid (C18:1(n-9)) and linolenic acid (C18:2), were significantly decreased, while the level of stearic acid (C18:0) was only slightly increased in the PTE (0.05 g/mL) treated group compared to the control group (Figure 1A). Thin-layer chromatography and gas chromatography (TLC/GC) analysis revealed that the percentage of triacylglycerols (TAG) in total lipids (TAG + PL, phospholipids) decreased from 50.05% in the control group to 44.5% in the PTE treated group (Figure 1B), nearly a 5.5% reduction in fat storage (Figure 1C). In addition, PTE reduced the lipid droplet size stained by Nile Red after fixation (Figure 1D). The average lipid droplet size was 1.66 ± 0.58 um (*n* = 10) in the control group, while it was 1.30 ± 0.68 um (*n* = 10) in the PTE treated group (Figure 1E).

### 2.2. Transcriptome Results Analysis

To obtain a genomic view of genes expression by PTE, we carried out RNA-Seq, and detected 18,073 and 18,105 genes in 0.025 g/mL and 0.05 g/mL PTE treated groups, respectively (Supplementary data). To search for different expression genes between the control group and the PTE treated group, we used Cufflinks (Cufflinks Software v2.0.2, California, USA) and found out that the expression of 18 genes was distinctly downregulated in the 0.05 g/mL PTE treated group compared to the control group. Interestingly, the expression of only four genes was changed in the 0.025 g/mL PTE treated group, and all of them were found in the list of 18 genes (Table 1). The low concentration of PTE (0.025 g/mL) decreased the expression of *vit-4*, *vit-5, col-7*, and *col-62* genes (Table 1), while the high concentration of PTE (0.05 g/mL) reduced the expression of *col-7, -8, -62, -81, -126, -127,* and *-146,* as well as *vit-1* to *vit-5* genes (Table 1). Thus, the expression of *vit-4*, *vit-5, col-7*, and *col-62* was responsive to PTE in a dose-dependent manner.

As we mentioned above, since PTE is able to reduce fat accumulation, we asked which bioprocess is involved. Then, DAVID (Database for Annotation, Visualization and Integrated Discovery) and GO (Gene orthology) annotation were used for bioprocess exploration. Strikingly, our results showed that PTE mainly affected two biological processes, in which one is involved in nematode cuticle collagen, and the other is involved in lipid transport (Table 1). The vitellogenin gene family (*vit-1* to *vit-5*), which encodes yolk protein that provides essential nutrients to the developing embryo and has similarities to the human apolipoprotein B-100 precursor protein, was all downregulated by PTE. In addition, the expression of several other genes was also changed by PTE at 0.05 g/mL concentration.

### 2.3. Validation of RNA-Seq Results by Quantitative PCR (qPCR)

**Figure 2:**
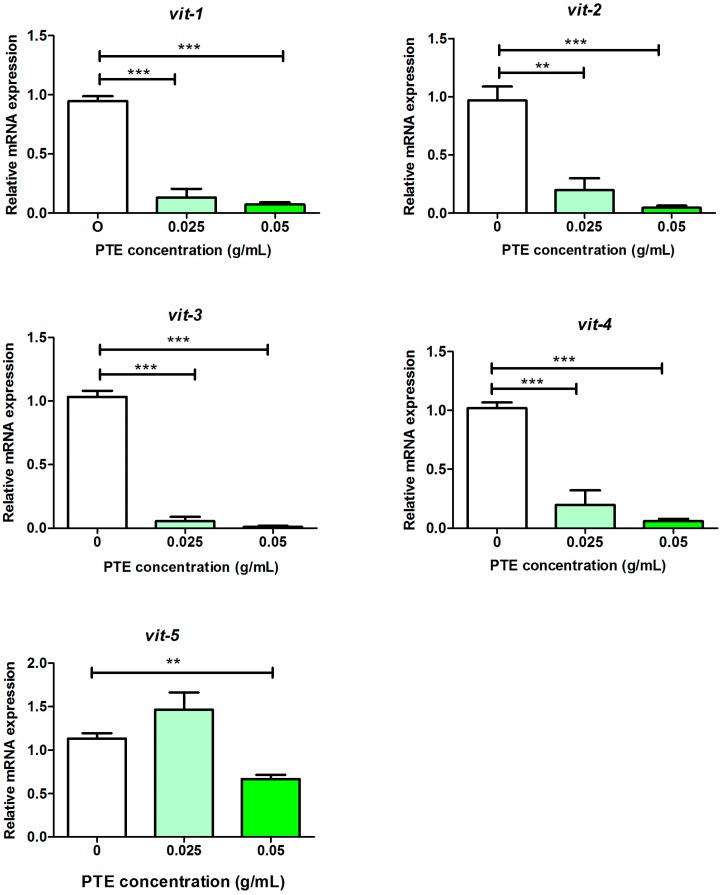
Relative mRNA expression of *vit-1, vit-2, vit-3, vit-4,* and *vit-5* by qPCR. Data are presented as mean ± SEM of four biological repeats. Significant differences between the control group and a specific PTE treated group, *** *p* < 0.001, ** *p* < 0.01.

Since the transcriptional expression of the vitellogenin genes (*vit-1* to *vit-5*) was downregulated by PTE, we then picked this gene family to validate the results of the transcriptome profile by using quantitative PCR (qPCR) analysis. Consistently, the expression of *vit-1, -2, -3,* and *-4* indeed decreased in a dose-dependent manner, while the expression of *vit-5* decreased only at high dosage of PTE (0.05 g/mL) (Figure 2). Therefore, transcriptional profile and qPCR results consistently showed that the expression of the *vit* gene family was definitely inhibited by PTE.

### 2.4. Mutants of Vitellogenin Genes are Resistant to PTE Reduced Fat Accumulation

**Figure 3:**
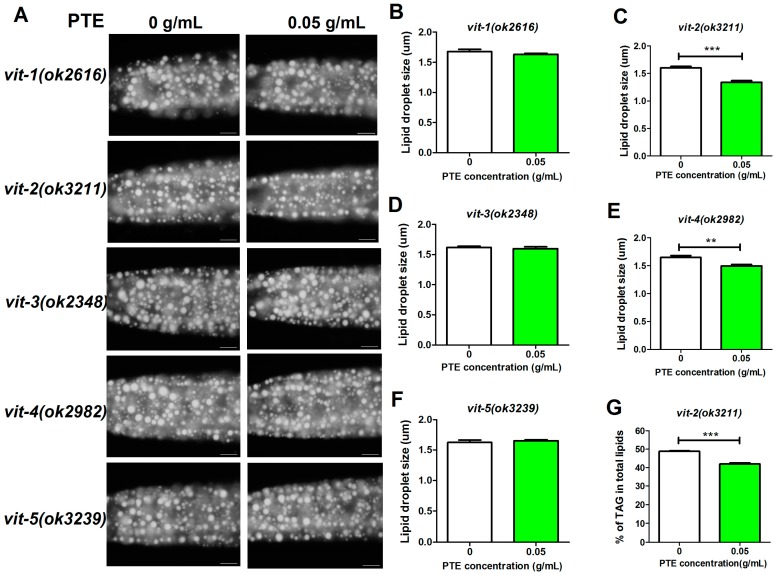
Nile Red staining of fixation and lipid droplets size of *vits* mutants treated by PTE. (**A**) Nile Red staining of late L4 worms treated with different concentrations of PTE. Presented animals, anterior is on right, and posterior is on left. Exposure time: 25 ms. Scale bar represents 20 μm; (**B**–**F**) quantification of lipid droplet size. Data are presented as ± SEM of 10 worms; and (**G**) percentage of TAG in total lipids (TAG + PL, phospholipids). Data are presented as mean ± SEM of four biological independent repeats. Significant differences between PTE treated group (0.05 g/mL) and the control group (0 g/mL), *** *p* < 0.001, ** *p* < 0.01.

Previous studies have demonstrated that vitellogenin proteins facilitate lipids to transport and store [30,31]. Therefore, we hypothesized that vitellogenin genes may play an important role in fat storage by PTE treatment. Among the mutants of the five *vit* genes, *vit-2(ok3211)* and *vit-4(ok2982)* displayed similar effects to N**_2_** when treated with PTE because the size of lipid droplet decreased (Figure 3A,C and E). Consistently, the TAG content of *vit-2(ok3211)* mutant quantitated by TLC/GC also decreased from PTE treatment (Figure 3G). Therefore, these two genes might not play roles in fat storage, although their transcriptional expression was inhibited under PTE treatment. In contrast, *vit-1(ok2616)*, *vit-3(ok2348)*, and *vit-5(ok3239)* mutants were unresponsive to PTE treatment, since their fat accumulation indicated by Nile Red of fixation and their lipid droplet size was not affected by PTE (Figure 3A,B,D and F). Collectively, these data suggest that the reduction of fat accumulation by PTE probably depends on the activity of VIT-1, VIT-3 and VIT-5.

### 2.5. Discussion

Pu-erh tea, one of the most popular beverages in the world, has been taken into consideration in recent years because of its potential health benefits. Animal studies have showed that Pu-erh tea has the capacity to lower total cholesterol, low-density lipoprotein cholesterol (LDL-C), and triglycerides levels [16]. Consistent with our previous study [22], PTE indeed decreased fat accumulation and lipid droplet size in *C. elegans*, suggesting a conserved role of anti-obesity by Pu-erh tea across metazoan. The constitutions of Pu-erh tea are complicated, which generally contain negligible amounts of ordinary catechins, but high levels of gallic acid [32]. Gallic acid is a well-known natural product that has anti-oxidative and anti-cancer function, and is also shown with anti-obesity activity in mice fed on a high-fat diet [33,34,35]. However, our previous study did not find a fat reduction effect by gallic acid in *C. elegans* [22], suggesting distinct roles of gallic acid between different organisms. Therefore, the bioactive compound still needs further investigation.

**Figure 4:**
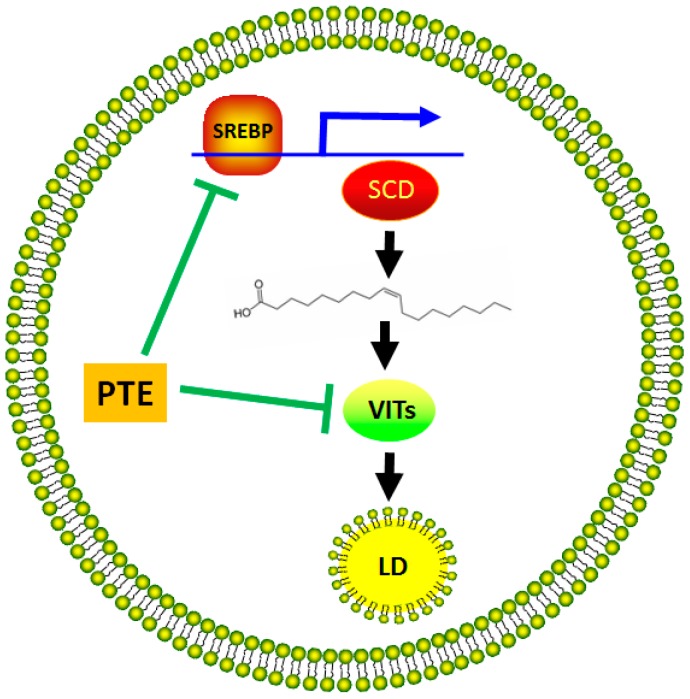
A proposed model to explain the underlying mechanisms of the Pu-erh tea anti-obesity effect. Pu-erh tea downregulates the expression of transcription factor SREBP (sterol regulatory element binding protein), which then reduces the transcriptional expression of SCD (stearoyl-CoA desaturase) that converts saturated fatty acids to monounsaturated fatty acids, leading to a reduction of de novo synthesis of fatty acids [22]. Meanwhile, Pu-erh tea also inhibits the expression of *vits* to reduce lipid transport to lipid droplets (LD) for storage, thereby leading to fat reduction. Please explain the yellow ball and wave line. The outside yellow ball indicates the double phophoslipid memebranes of cell, and the inside small yellow ball indicates intracellular lipid droplet wrapped by monolayer phophoslipid. The wave line indicates a monounsaturated fatty acid.

RNA-Seq technique or proteomic analysis has been used to investigate the molecular mechanisms of the lipid-lowering effect of nature products [36,37]. It remains unknown how Pu-erh tea regulates the global genes expression at genomic level. To the best of our knowledge, we might be the first to use the high-throughput RNA-Seq technique to study the influence of Pu-erh tea on fat reduction in model organism *C. elegans*. By comparative analysis of transcriptome profiles, we found that PTE downregulates the transcriptional expression of *vits* family in a dose-dependent manner. Six diverse *vit* genes encoding vitellogenins are present in *C. elegans* [38], and they possess a high sequence homology [39]. VIT family members have important functions in transporting lipids from intestine to oocyte, facilitating yolk formation and embryonic development. Previously, we showed that VIT-2 is required for fat accumulation induced by iron-overload in *C. elegans* [31]. In addition, some signal pathways and genes have been found to be responsible for the anti-obesity effect of Pu-erh tea [19,22], of which we previously revealed that PTE downregulates the expression of the transcription factor SREBP and its main target SCD, which are involved in the de novo synthesis of fatty acids, to reduce fat accumulation in worms. Based on these results, we propose here a model to explain the possible mechanisms of the anti-obesity effect of Pu-erh tea (Figure 4). Pu-erh tea not only represseses the de novo synthesis of fatty acids by downregulation of the expression of SREBP and SCD, but also inhibits the expression of *vits* to reduce transport of lipids to lipid droplets for storage, thereby leading to fat reduction.

## 3. Materials and Methods

### 3.1. Production of the Aqueous Extract of Pu-Erh Tea

Pu-erh tea (Brand number GBT/22111), produced in 2011, was purchased from LongRun Tea Industry (Kunming, Yunnan, China). The water extract of Pu-erh tea was prepared followed our reported method [22]. Briefly, Pu-erh tea (100 g) was soaked in 500 mL boiling water for 20 min, and the water extract was centrifuged 10,000 r/min for 10 min at room temperature. The water tea was filtered and added to the nematode growth medium (NGM) to the concentration of 0 g/mL, 0.025 g/mL, and 0.05 g/mL.

### 3.2. Culture Conditions and Worm Strains

The wild-type N**_2_** strain was used as a control for all experiments. Genetic mutants used for this study include: *vit-1(ok2616)X, vit-2(ok3211)X, vit3(ok2348)X, vit4(ok2982)X, vit5(ok3239)X*. All strains were grown on NGM plates (Kai Rui Da, Jiangsu, China) with or without Pu-erh tea water extract (PTE) seeded with *Escherichia coli* OP50. Worms were raised at 20 °C as previously described [22].

### 3.3. Samples Collection and Sequencing

Worms treated with different concentrations of PTE (0 g/mL, 0.025 g/mL, 0.05 g/mL) were collected for transcriptsome analysis. Briefly, animals were washed off of plates at the age of late L4 and young adult. Total RNA was extracted with Trizol (Transgene, Beijing, China) according to the manufacture’s protocols, and sequenced by using an Illumina HiSeq2000 instrument (Illumina, San Diego, CA, USA). The library preparation and sequencing were performed as previously described [40].

### 3.4. Nile Red Staining of Fixation

Late L4s and young adult worms were washed off of the NGM plates with M9 and fixed by paraformaldehyde. After freezing three times, they were stained with Nile Red as previously described [41,42].

### 3.5. Lipid Extraction and Analysis

Young adult worms with 2–3 eggs (>50,000) treated with different concentrations of PTE were washed off of NGM plates. Then, the worms were frozen immediately in liquid nitrogen and stored at −80 °C in the freezer for further study. Thin-layer chromatography (TLC) was performed to separate TAGs and phospholipids (PL); fatty acid compositions were analyzed by gas chromatography (GC, Agilent, Santa Clara, CA, USA) as previously described [22,41,42,43,44]. Briefly, lipids were extracted overnight at 4 °C with chloroform:methanol (1:1). The extract was washed with 0.2 M H_3_PO_4_, 1 M KCl, and lipids were recovered in the chloroform phase and dried under argon. Neutral lipids were separated by thin layer chromatography on silica gel plates (Huang Hai, Shandong, China). Triacylglycerol and phospholipid fractions were scraped for fatty acid methyl ester derivatization and analyzed by gas chromatography. Fatty acid composition was analyzed with an Agilent 7890A gas chromatographer equipped with a 15 m × 0.25 mm × 0.25 μm DB-WAX column (Agilent). C15:0 was used as a standard for quantitation.

### 3.6. RNA Isolation and qPCR Analysis

Total RNA was isolated with over 1000 young adult worms using TransZol Up (Code#ET111–01, TransGen Biotech, Beijing, China), and reversed to cDNA using Prime Script RT reagent Kit (Cat#RR047A, Takara Bio Inc. Kusatsu, Japan). SYBR1 × green mix (Trans Start TipTop Green qPCR SuperMix, Cat#AQ141, TransGen Biotech, Beijing, China) was used for the qPCR reaction. The real-time amplification was monitored by ABI 7900HT analyzer (Applied Biosystems, Foster City, CA, USA). Relative expression of genes was calculated according to the ΔΔCt method [42]. β-Actin was used as the reference gene.

## 4. Conclusions

In this work, we revealed that Pu-erh tea reduces fat accumulation primarily through repression of the expression of the *vit* family by RNA-Seq in the transcriptional profile. We also demonstrated that *C. elegans* could be a useful animal model to investigate the anti-obesity effect of a drug or therapy.

## Figures and Tables

**Table 1:** Genes that showed differencially expression treated by different concentrations of PTE.

Gene ID	Gene Symbol	Biological Function	Fold Change		*p*-Value	
			0.025 g/mL	0.05 g/mL	0.025 g/mL	0.05 g/mL
XLOC_000825	*col-7*	Nematode cuticle collagen	(−)17.21	(−)86.04	2.6 × 10^−6^	2.6 × 10^−^^10^
XLOC_010817	*col-8*	(S)		(−)68.00		2.11 × 10^−6^
XLOC_002700	*col-62*	(S)	(−)15.32	(−)45.24	3.02 × 10^−6^	2.32 × 10^−^^8^
XLOC_007760	*col-81*	(S)		(−)9.33		2.93 × 10^−6^
XLOC_014356	*col-126*	(S)		(−)19.12		5.21 × 10^−6^
XLOC_021979	*col-127*	(S)		(−)19.12		5.21 × 10^−6^
XLOC_028294	*col-146*	(S)		(−)13.27		2.88 × 10^−6^
XLOC_035276	*vit-1*	Lipid transport		(−)6.53		2.79 × 10^−^^5^
XLOC_034756	*vit-2*	(S)		(−)9.68		1.56 × 10^−^^5^
XLOC_037318	*vit-3*	(S)		(−)7.79		1.72 × 10^−^^5^
XLOC_037317	*vit-4*	(S)	(−)8.16	(−)13.68	5.83 × 10^−6^	9.06 × 10^−^^8^
XLOC_034472	*vit-5*	(S)	(−)18.37	(−)73.18	1.81 × 10^−^^9^	2.86 × 10^−^^14^
XLOC_034954	C45B2.1	Imported		(−)19.39		1.55 × 10^−^^5^
XLOC_028510	D1054.11	(S)		(−)14.58		4.25 × 10^−^^5^
XLOC_005888	K10H10.4	Protein binding		(−)31.48		5.45 × 10^−6^
XLOC_006818	*dct-5*	Defence responce		(−)34.82		5.74 × 10^−6^
XLOC_031134	*abu-7*	Protect organism		(−)12.82		4.41 × 10^−^^5^
XLOC_000825	*grl-4*	Structural molecule activity		(−)13.39		2.27 × 10^−6^

Note: (S) indicated same biological function as the first member of family.

## Supplementary Materials

The following are available online at http://www.mdpi.com/1420-3049/21/10/1379/s1. Data are presented as mean ± SEM. Statistical analysis performed included *t*-test or analysis of variance (ANOVA) followed by LSD (The least significant difference test) multiple comparisons using SPSS 20.0 (IBM SPSS Statistics, Armonk, NY, USA). All figures were made by GraphPad Prism 5 (GraphPad Software, La Jolla, CA, USA).
